# Optimal encephalitis/meningitis roadmap via precise diagnosis and treatment (IMPROVE): a study protocol for a randomized controlled trial

**DOI:** 10.1186/s12879-021-06943-6

**Published:** 2022-01-08

**Authors:** Yi Zhang, Haocheng Zhang, Bo Deng, Ke Lin, Lei Jin, Xiaoni Liu, Yanlin Zhang, Xiaohua Chen, Yanliang Zhang, Shengjia Lu, Heqing Huang, Qiujing Wang, Tingting Feng, Weifeng Zhao, Qun Xue, Renfang Chen, Jingbo Zhang, Xiaoyan Qian, Lanlan Chen, Jingwen Ai, Xiangjun Chen, Wenhong Zhang

**Affiliations:** 1 Department of Infectious Diseases, National Medical Center for Infectious Diseases, Shanghai Key Laboratory of Infectious Diseases and Biosafety Emergency Response, Huashan Hospital, Fudan University, Shanghai, 200040 China; 2 Department of Neurology, Huashan Hospital, Fudan University, Shanghai, China; 3 Department of Neurology, The Second Affiliated Hospital of Soochow University, Suzhou, Jiangsu Province China; 4 Department of Infectious Diseases, Shanghai Sixth People’s Hospital Affiliated to Shanghai JiaoTong University, Shanghai, China; 5 Department of Infectious Diseases, Nanjing Hospital of Chinese Medicine Affiliated to Nanjing University of Chinese Medicine, Nanjing, Jiangsu Province China; 6 Department of Neurology, Tongde Hospital of Zhejiang Province, Hangzhou, Zhejiang Province China; 7 Department of Infectious Diseases, Zhuji People’s Hospital of Zhejiang Province, Shaoxing, Zhejiang Province China; 8 Department of Infectious Diseases, Zhoushan Hospital of Zhejiang Province, Zhoushan, Zhejiang Province China; 9 Department of Infectious Diseases, The First Affiliated Hospital of Soochow University, Suzhou, Jiangsu Province China; 10 Department of Neurology, The First Affiliated Hospital of Soochow University, Suzhou, Jiangsu Province China; 11 Department of Infectious Diseases, Wuxi No.5 People ‘s Hospital, Wuxi, Jiangsu Province China; 12 Department of Neurology, Blue Cross Brain Hospital, Shanghai, China; 13 Department of Neurology, The first people’s hospital of Kunshan, Suzhou, Jiangsu Province China; 14 Department of Neurology, Northern Jiangsu People’s Hospital, Yangzhou, Jiangsu Province China

**Keywords:** Encephalitis, Meningitis, Multicenter, Randomized trial, Diagnosis, Treatment

## Abstract

**Background:**

Encephalitis/meningitis brings a heavy disease burden, and the origin of disease remains unknown in 30–40% of patients. It is greatly significant that combinations of nucleic acid amplification and autoimmune antibody testing improves the diagnosis and treatment of encephalitis/meningitis. Moreover, though several diagnostic methods are in clinical use, a recognized and unified diagnosis and treatment process for encephalitis management remains unclear.

**Methods:**

IMPROVE is a multicenter, open label, randomized controlled clinical trial that aims to evaluate the diagnostic performance, applications, and impact on patient outcomes of a new diagnostic algorithm that combines metagenomic next-generation sequencing (mNGS), multiplex polymerase chain reaction (PCR) and autoimmune antibody testing. The enrolled patients will be grouped into two parallel groups, multiplex PCR test plus autoimmune antibody group (Group I) or the mNGS plus autoimmune antibody group (Group II) with a patient ratio of 1:1. Both groups will be followed up for 12 months. The primary outcomes include the initial time of targeted treatment and the modified Rankin scale score on the 30th day of the trial. The secondary outcomes are the cerebrospinal fluid index remission rate on the 14th day, mortality rate on the 30th day, and an evaluation of diagnostic efficacy. The two groups are predicted to comprise of 484 people in total.

**Discussion:**

To optimize the roadmap of encephalitis/meningitis, precise diagnosis, and treatment are of great significance. The effect of rapid diagnosis undoubtedly depends on the progression of new diagnostic tests, such as the new multiplex PCR, mNGS, and examination of broad-spectrum autoimmune encephalitis antibodies. This randomized-controlled study could allow us to obtain an accurate atlas of the precise diagnostic ability of these tests and their effect on the treatment and prognosis of patients.

*Trial registration* ClinicalTrial.gov, NCT04946682. Registered 29 June 2021, ‘Retrospectively registered’, https://clinicaltrials.gov/ct2/show/NCT04946682?term=NCT04946682&draw=2&rank=1

## Background

Encephalitis/meningitis are defined as encephalopathy with associated seizures, neurological dysfunction and focal neurological deficits; there are a wide variety of possible infectious and autoimmune mechanisms [1]. The annual incidence of encephalitis is about 12.6/100,000. Encephalitis/meningitis has a heavy disease burden, and 5–26% of patients suffer from neurological sequelae with a mortality rate of up to 5–10% [1]. Between 40 and 50% of cases with known causes are attributable to infections and of these, viruses are the most common cause, followed by bacteria. In viral infections, *Japanese encephalitis virus* is the most common epidemic virus, and *herpes simplex virus* (HSV) is the most commonly detected sporadic pathogen [2]. Autoimmune encephalitis (AE) accounts for approximately 20–30% of encephalitis/meningitis cases.

The timely identification of causative agents is critical for the administration of effective treatment for infectious encephalitis/meningitis. Multiplex polymerase chain reaction (PCR) sequencing, such as the FilmArray meningitis/encephalitis and Xpert MTB/RIF Ultra panels, are able to improve diagnostic capability in a limited spectrum of pathogens, however this still does not meet clinical needs. Therefore, there is still a need for rapid detection methods with a wide pathogen spectrum in order to improve the diagnostic process of infectious encephalitis [3–6]. In recent years, metagenomic next-generation sequencing (mNGS) has been shown to have great advantages in the diagnosis of infectious diseases, especially in bloodstream, respiratory tract, and central nervous system (CNS) infections. It is proposed that the diagnosis rate of CNS infections can be improved by approximately 25% with the application of mNGS [7]. This process features a short turnaround time, unbiased detection and dynamic surveillance [8]. Application of mNGS may improve and optimize diagnostic strategies for infectious encephalitis/meningitis.

The rapid development of cell-based assays (CBAs), tissue-based assays (TBAs) and B cell repertoire analyses accelerated the discovery of novel autoantibodies and their applications for the diagnosis of AE [9, 10]. CBAs are commonly used in clinical practice, but can only detect a narrow spectrum of autoantibodies that are targeted to currently known antigens. On the other hand, TBAs can facilitate the discovery of unknown autoantibodies [11]. Therefore, the combination of broad-spectrum CBAs and TBAs could be a powerful tool in the diagnosis of autoantibody-associated encephalitis. However, the diagnosis of AE requires the simultaneous exclusion of other possible diseases, thus, it is of great importance to combine nucleic acid amplification testing for infectious agents with autoimmune antibody tests to improve the diagnosis and treatment of encephalitis/meningitis [12, 13]. Moreover, though several diagnostic methods have been used, a recognized and unified diagnosis and treatment process for encephalitis management remains unclear.

Therefore, we propose a multicenter, open label, randomized controlled clinical trial to evaluate the diagnostic performance of a new diagnostic algorithm which combines mNGS and autoimmune antibodies analysis. The impact on the outcomes of the patients enrolled will also be assessed. We hope to develop an optimized process for the diagnosis and treatment of encephalitis/meningitis which will result in more effective treatments.

## Methods

### Study design

**Fig. 1:**
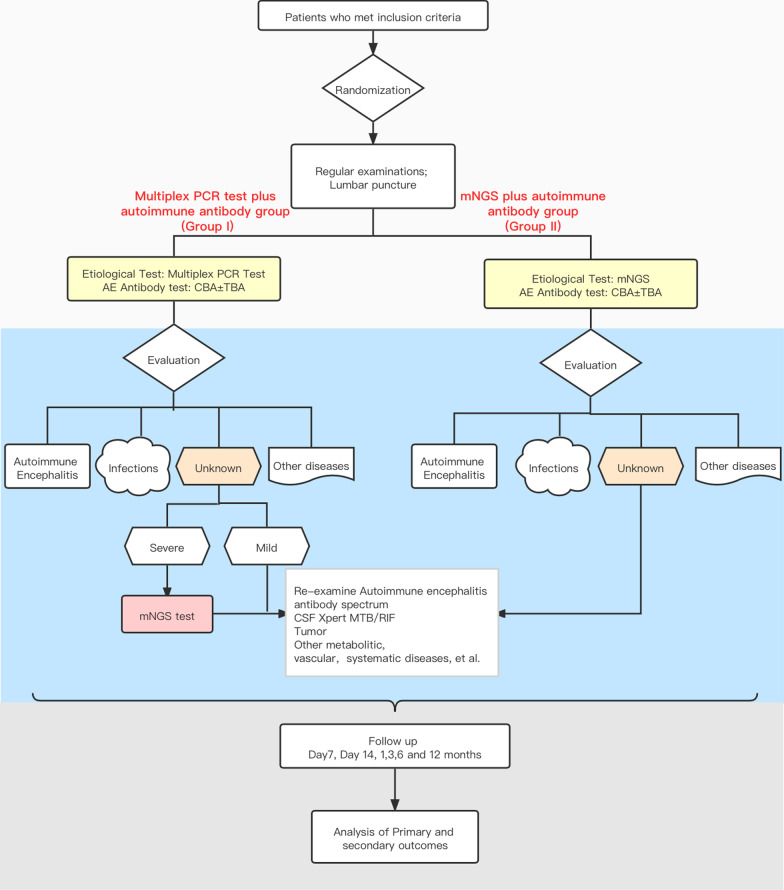
IMPROVE study outline

This study is a multicenter open label, randomized controlled clinical trial of non-inferiority with two arms recruiting patients with suspected encephalitis/meningitis. The enrolled patients will be grouped into two parallel groups with a ratio of 1:1, those whose diagnoses were achieved through multiplex PCR tests plus autoimmune antibody analyses (Group I), and those being assessed through the use of mNGS plus autoimmune antibody testing (Group II). The detailed study schematic is shown in Fig. 1.

### Ethical and confidential considerations

The study protocol and informed consent forms have been approved by the Huashan Hospital Ethical committee (protocol ID: 2021-KY451). The clinician should obtain face to face informed consent during an interview before enrollment. All patients will provide written informed consent.

### Site selection

This trial will be led by the Huashan Hospital Affiliated to Fudan University. The recruiting-cooperative units were distributed over three provinces in China: hospitals in Shanghai include Shanghai Sixth People’s Hospital Affiliated to Shanghai JiaoTong University and Blue Cross Brain Hospital; Nanjing Hospital of Chinese Medicine Affiliated to Nanjing University of Chinese Medicine, The First Affiliated Hospital of Soochow University, The Second Affiliated Hospital of Soochow University, Wuxi No.5 People ‘s Hospital, The first people’s hospital of Kunshan, and Northern Jiangsu People’s Hospital in Jiangsu; and hospitals in Zhejiang include Zhoushan Hospital of Zhejiang Province, Zhuji People's Hospital of Zhejiang Province and Tongde Hospital of Zhejiang Province. A contract research organization will take responsibility for the quality control of these cooperative centers for 1 year.

### Study flowchart and clinical evaluation of enrolled patients

Adult patients who meet the inclusion criteria will be eligible (Table 1). Eligible patients with suspected encephalitis/meningitis will be included after signing the informed consent forms. As outlined above, the participants will be randomly assigned in a 1:1 ratio to Group I or Group II. The randomization is performed through the online central randomization system stratified by the study sites (Clinflash, China). To facilitate autoimmune encephalitis antibody tests and molecular etiological examinations, 13 ml of peripheral blood and 8 ml of cerebrospinal fluid (CSF) will be collected. The first screen will include basic patient characteristic, as well as additional information such as present history, functional assessments (modified Rankin Scale [mRS], Glasgow outcome score [GOS] and EuroQol-5 Dimension (EQ-5D)), physical examinations, cranial magnetic resonance imaging (MRI), blood examinations including routine blood tests, C-reactive protein, synchronous glucose, D-dimer, *Treponema pallidum* particle agglutination assay/rapid plasma reagin, thyroid hormones and thyroid-related antibodies; CSF pressure, biochemical and routine tests; and etiological examinations of CSF including latex agglutination test, ink stain, and bacterial, fungi and tuberculosis cultures. Electroencephalograms are to be performed according to clinical necessity.

**Table 1:** Inclusion and exclusion criteria

Inclusion criteria
• Aged from 18 to 80 years old
• Without clear diagnosis
• Without effective antimicrobial therapy
• The suspected meningitis/encephalitis patients should meet the ① or ② items as following:
① Suspected meningitis: Rapid onset fever (> 38.5 °C). with at least one of the three manifestations as following:
(1) Neck stiffness
(2) Alteration of consciousness
(3) Other signs indicating meningitis, for example, meningeal irritation sign
② Suspected encephalitis: Altered consciousness because of unidentified reasons that persisted for longer than 24 h, including lethargy, irritability, or a change in personality, and have at least one of these manifestations:
(1) Fever or history of fever (≥ 38 °C, oral temperature) during the presenting illness
(2) Seizures not explained by a previously known seizure disorder
(3) CSF pleocytosis (white blood cell count of more than 4 cells per μl)
(4) Electroencephalographic (EEG) findings indicative of encephalitis
(5) Abnormal results of neuroimaging (CT or MRI) suggestive of encephalitis
• Have an identifiable address and resided in the area during treatment
• Willing to participate in the treatment and follow-up and able to provide informed consent (if subject is illiterate, legal guardian sign or consent is required)
• Willing to comply follow-up procedures
Exclusion criteria (≥ 1 items)
**•** Lactation or pregnancy
• Fail to comply follow-up or treatment
• Researchers consider it is unsafe for participant
• No enough blood or CSF samples

For both Group I and II, patients who have positive pathogenic results are considered to have the infectious disease, and those with positive autoimmune encephalitis antibodies are classified as having autoimmune encephalitis; other diseases are confirmed by their corresponding gold standard tests and classified as “other diseases” and the remaining subjects are categorized as being of unknown origin.

Patients with infections of unknown origin are divided into severe encephalitis/meningitis and mild encephalitis/meningitis based on their clinical manifestations. Patients with severe encephalitis/meningitis should meet at least one of five criteria: (1) Disturbance of consciousness: GOS < 8; (2) New onset of epilepsy or epileptic status; (3) Respiratory failure requiring mechanical ventilation and support; (4) Circulatory failure with vasoactive drug use; (5) Complicated multiple organ failure requiring intensive care unit monitoring and treatment. If a patient in Group I develops severe encephalitis/meningitis with an unclear cause after empirical treatment is given, mNGS could be conducted between the 3rd and 14th day of the trial.

Several additional examinations are recommended for patients with infections of unknown origin in both Group I and II. These analyses include CSF Xpert TB/RIF, extended antibody spectrum and paraneoplastic antibody screening, and examination for tumors, congenital, metabolic and cerebrovascular diseases; poisoning, and so on. Targeted treatment should be applied according to test results.

Both groups will be followed up for 12 months and the final diagnosis will be made by doctors at the treating hospital and by physicians of Huashan Hospital which is affiliated with Fudan University.

### Diagnostic methods

We will provide either multiplex PCR tests or mNGS (including examination of both DNA and RNA) for microbiological examinations in this study. According to recommendations, we will include HSV types 1 and 2; *Varicella zoster virus*, *Enteroviruses*, *Neisseria meningitidis* and *Streptococcus pneumoniae* [14]. The autoimmune antibody spectrum will be established over several admission days as the disease progresses (Table 2).

**Table 2:** The detailed autoimmune antibody spectrum of each sampling day

Admission days	Samples	Methods	Antibody spectrum
Day 0	Blood and CSF	CBA and TBA	NMDAR, GABABR, LGI1, Caspr2, AMPAR1, AMPAR2, GAD, MOG
Day 7	Blood and CSF	CBA and paraneoplastic antibody OR TBA	NMDAR, LGI1, GABABR, CASPR2, AMPAR1, AMPAR2, IgLON5, DPPX, GAD65, mGluR5, GlyR, D2R Paraneoplastic antibody: Hu, Yo, Ri, CV2(CRMP5), Ma1, Ma2, Amphiphysin, SOX1, Tr (DNER), Zic4, GAD65, PKCγ, Recoverin, Titin
Day 14	Blood and CSF	CBA	MDAR, GABABR, LGI1, Caspr2, AMPAR1, AMPAR2, GAD, MOG
Day 15–60	Blood and CSF	CBA ± TBA	MDAR, GABABR, LGI1, Caspr2, AMPAR1, AMPAR2, GAD, MOG
1/3/6/12 months	Blood	CBA	Re-examinations for autoimmune-positive patients

### Duration of follow-up

Follow up will take place on the 7th and 14th day and in the 3rd, 6th and 12th month of the trial. Follow-up examinations will mainly consist of clinical evaluation, physical examination, routine blood examinations including routine blood parameters, C-reactive protein, D-dimer, and synchronized glucose; CSF examinations such as pressure, biochemical and routine tests; nervous system analysis scores including mRS, GOS Score and EQ-5D; extra autoimmune encephalitis antibody tests and molecular etiology examination.

According to these examinations, if symptoms persist or the condition of the participant is worse between the 15th and 60th day, we may reperform additional autoimmune encephalitis antibody tests and molecular etiology examinations (mNGS test).

In summary, detailed follow up examination will proceed in the following order; clinical evaluation, physical examination and nervous system analysis scores should be evaluated on the 7th and 14th day of the trial. In addition, a lumbar puncture and routine blood examination should also be conducted on these days, and CSF will be subjected to autoimmune encephalitis antibody tests (Table 2). Assessments in the 3rd and 12th month will involve clinical evaluation, physical examination, and nervous system analysis scores.

### Sample size calculation

The predicted sample size was calculated using the PASS 11 software. Assuming hazard ratio (HR) = 1.48 and θ = 1, and according to previous research, pE = 0.7, PA = 0.5, each group requires 230 samples. Assuming a 5% drop-out rate in follow-up, each group needs 230 × 1.05 = 242 people; therefore, the total sample size of the two groups combined is calculated to be 484 people.

### Data collection and quality management

Several quality assurance practices should be established and undertaken both before and during project implementation. The design of the project (Fig. 1) was based on previous studies undertaken by both the Department of Infectious Disease and the Department of Neurology of the Huashan Hospital affiliated with Fudan University. All relevant personnel involved in the project, including clinicians, laboratory, and project management personnel have received uniform training; which includes theoretical explanations and internship operations. Furthermore, we have devised a number of inclusion standards and involved clinicians have undergone specialized training on relevant topics and become familiar with the principles and standards of case inclusion. To ensure that cases are representative, every case that meets the inclusion criteria during the implementation of the project must be included and cannot be selected or omitted. Each case will be reviewed by the subject person in charge of Huashan Hospital affiliated with Fudan University.

Data collection also requires quality management of all aspects. Clinicians responsible for filling in the subject information forms have received uniform training on the subject. They will have a clear understanding of the questions in the information sheet before formally asking the participants and will be able to fill in the relevant content accurately according to the requirements in the uniformly designed case report form (CRF). The clinician must ask the participants the questions face to face while completing the form, rather than making assumptions or filling it out by recollection afterwards.

To ensure the authenticity and objectivity of the subject material, all relevant forms should be properly stored. All registration books and form cards must be kept in a suitable place that is safe from flooding, burning, tearing, smearing, and so on. A person should be dedicated to ensure that all information can be accessed after the on-site investigation.

In order to ensure that each center conducts the research in strict accordance with the design plan, and that the data is true and credible, the project leader will be supervised by the Encephalitis/Meningitis Research Group of Huashan Hospital affiliated with Fudan University, once every 2 months. Supervisors will be assigned by the Huashan Hospital affiliated with Fudan University.

### Adverse event management

Potential adverse events and their management have been evaluated. In this study, an additional 13 ml of peripheral blood and 8 ml of CSF will be drawn during routine clinical examinations in the enrolled participants No additional operations or costs are required. However, the process to collect extra blood and CSF, medical history and other information may cause psychological discomfort to the participants. There might also be risks, such as short-term pain and local bruising during blood withdrawal and lumbar puncture. Some people may experience mild dizziness, and in extremely rare occasions, an infection at the needle site may occur. Collection of extra CSF during lumbar puncture may potentially cause mild headaches and backaches following this process.

To further evaluate and avoid adverse events, we will record these events and they will be attended to by physicians. If a patient has any psychological discomfort during the collection of their medical history, the researcher will provide timely comfort and notify the designated personnel of the research group to record and evaluate this event within 2 h. If verbal comfort is ineffective for any participant, such as in the cases of depression or mania and so on, a psychologist will be required to conduct an evaluation and psychological counseling within 12 h, and this will be recorded by the designated personnel from the research team. Fluid infusion may be administered to participants with mild headaches and backaches after lumbar puncture. In the event of unexpected injury or loss related to this study, the patient will receive appropriate treatment or compensation. All adverse events in this clinical study will be recorded in detail by a dedicated person. Any serious adverse events will be handled and recorded by a dedicated person and reported to the ethics committee and relevant departments timely.

### Assessment and analysis of outcomes

The primary and secondary outcomes between Group I and Group II will be compared. Primary outcomes are the initial time of targeted treatment and mRS score on the 30th day whilst secondary outcomes include CSF index remission rate on the 14th day, mortality rate on the 30th day, and the evaluation of diagnostic efficacy. Through these assessments, we may explore the best diagnostic algorithm for encephalitis/meningitis, and finally determine its optimal treatment.

The results for the trial outcomes will be analyzed through the following methods. First, we will review whether the enrolled cases meet the selection and exclusion criteria, and whether the actual number of enrolled, excluded and dropped cases in each center are accounted for at the end of the study; we will then perform a comparability analysis. Data relating to demographic characteristics, general and baseline (before the test) conditions will be compared between the two groups. The statistical analyses to be employed include two-sided t-tests, Wilcoxon tests, and chi-square tests—which will compare the outcomes between the two groups. A P value ≤ 0.05 was considered statistically significant. Excel was adopted for the management of data and the performance of statistical analysis after verification. SPSS 11.0 and PRISM 9.0 were the software used for statistical analyses.

### Confidentiality

Participants’ personal information will be confined to only that which is required for the outcome evaluation of the research regimen, in accordance with applicable privacy and confidentiality regulations. Paper papers containing participant information will be kept in a secure location in cooperative hospitals. The digital documents will be saved in password-protected folders. Only authorized people will have access to the study documents.

## Discussion

To optimize the roadmap of encephalitis/meningitis, precise diagnosis, and treatment are of great significance. Although an etiology surveillance study was performed by the California Encephalitis Project (CEP) [15], follow-up was not continued. Moreover, the diagnosis and treatment roadmap of encephalitis/meningitis remains unclear. Thus, this study applies novel immunological and molecular diagnostic methods, including multiplex PCR tests, mNGS and a broadened spectrum of autoimmune brain antibodies to improve the algorithm of diagnosis and treatment for encephalitis/meningitis.

CEP was launched in 1998 to identify the etiologies of encephalitis [15]. This project enrolled over 4000 cases between 1997 and 2010, and represents the largest study of patients with encephalitis to date [16]. Despite the rigorous diagnostic testing algorithm in use at that time, the underlying causes in approximately 50% of cases were still undetermined. In 2005, Granerod et al. conducted a population-based prospective study on the etiology of encephalitis in England [17]. This study found that the proportion of unknown etiology could be reduced with extensive testing, such as PCR, serological tests, and antibody assays; however, the proportion of patients with an unknown etiology was still higher than for any specific identified cause. Both of the aforementioned studies were conducted before mNGS and extensive autoantibody tests became available.

Highly sensitive rapid diagnostic tests might be useful for the detection of infections of the CNS to rule out potentially fatal conditions and indicate appropriate, targeted treatment of the patient. The effect of rapid diagnosis undoubtedly depends on the progression of new diagnostic tests, such as the new multiplex PCR, mNGS test, and so on.

Combining several PCR diagnostic tests into a single device capable of detecting analytes of interest was the optimized method to detect specific pathogens. However, devices with a fixed combination of tests are often more costly and are less adaptable. Therefore, the creation of a proper pathogen cassette able to detect the most common infectious diseases at a relatively low cost is a difficult task. Furthermore, quality assurance of multiplexed tests is not made easier by combining them in a single device. The pathogen cassettes made for this study include the most common pathogens that can cause encephalitis/meningitis syndromes; we attempted to prove that such cassettes can act as a quick and cheap diagnostic method for 41.0–58.5% [14, 18, 19] of infectious diseases of the CNS.

Our previous studies have reported satisfactory diagnostic performances in CNS infections, and mNGS proved overall superior with an extra 30% pathogen detection rate [20] compared to cultures, therefore confirming its capability in the dynamic surveillance of specific pathogens [21]. Nevertheless, the clinical effectiveness of mNGS including prognosis, treatment, and so on, has not been studied systemically. Rapid progression of diagnostic methods has helped clinicians determine final diagnoses easily and accurately, but sometimes this process is expensive. A promising diagnostic algorithm must be cost-effective to be sustainable as it can result in unnecessary and costly referrals to higher levels of care. To date, we still lack an effective diagnostic algorithm for encephalitis/meningitis syndromes in addition to associated validation of tests in field settings and validated evidence-based algorithms.

Over the past decade, more than 15 new types of auto-immune antibodies associated with autoimmune encephalitis have been identified; this has extended our understanding of the roles of autoantibodies in encephalitis extensively, and has changed the landscape of etiology in encephalitis [9, 15]. Previously, our group identified several genetic factors in AE pathogenesis and found that ^18^F-fludeoxyglucose positron emission tomography and computed tomography could distinguish patients with anti-*N*-methyl-d-aspartate encephalitis that were triggered by different factors [22]. Based on previous studies, we developed a combination of mNGS and a broad spectrum of AE antibodies for CBA and TBA. This randomized-controlled study could allow us to obtain an accurate atlas of its precise diagnostic ability and its effect on the treatment and prognosis of patients.

This study also has several limitations. Though we used multiplex PCR or mNGS in diagnosing infectious encephalitis/meningitis in the central laboratory, regular cultures and essential antibody assays were performed in local hospitals. In some hospitals without sufficient treatment and testing capabilities, the pathogens could not be identified through these traditional methods. Additionally, the pathogen cassettes we made for this study only included the six most common pathogens that can cause 70–80% of encephalitis/meningitis syndromes according to previous research.

## Data Availability

This is a study protocol manuscript, not applicable.

## Abbreviations

AE: Autoimmune encephalitis
CBA: Cell-based assay
CEP: California Encephalitis Project
CNS: Central nervous system
CSF: Cerebrospinal fluid
GOS: Glasgow outcome score
HSV: Herpes simplex virus
MRI: Magnetic resonance imaging
mNGS: Metagenomic next-generation sequencing
mRS: Modified Rankin scale
PCR: Polymerase chain reaction
TBA: Tissue-based assay
CRF: Case report form
HR: Hazard ratio
